# Finasteride induced depression: a prospective study

**DOI:** 10.1186/1472-6904-6-7

**Published:** 2006-10-07

**Authors:** Babak Rahimi-Ardabili, Ramin Pourandarjani, Peiman Habibollahi, Amir Mualeki

**Affiliations:** 1 Clinical Pharmacy Laboratory, Drug Applied Research Center, Tabriz University of Medical Sciences, Tabriz, Iran; 2 Sina's clinic of Dermatology, Tabriz University of Medical Sciences, Tabriz, Iran; 3 Faculty of Medicine, Tehran University of Medical Sciences, Tehran, Iran

## Abstract

**Background:**

Finasteride is a competitive inhibitor of 5 alpha-reductase enzyme, and is used for treatment of benign prostatic hyperplasia and androgenetic alopecia. Animal studies have shown that finasteride might induce behavioral changes. Additionally, some cases of finasteride-induced depression have been reported in humans. The purpose of this study was to examine whether depressive symptoms or anxiety might be induced by finasteride administration.

**Methods:**

One hundred and twenty eight men with androgenetic alopecia, who were prescribed finasteride (1 mg/day) were enrolled in this study. Information on depressed mood and anxiety was obtained by Beck Depression Inventory (BDI), and Hospital Anxiety and Depression Scale (HADS). Participants completed BDI and HADS questionnaires before beginning the treatment and also two months after it.

**Results:**

Mean age of the subjects was 25.8(± 4.4) years. At baseline, mean BDI and HADS depression scores were 12.11(± 7.50) and 4.04(± 2.51), respectively. Finasteride treatment increased both BDI (p < 0.001) and HADS depression scores significantly (p = 0.005). HADS anxiety scores were increased, but the difference was not significant (p = 0.061).

**Conclusion:**

This preliminary study suggests that finasteride might induce depressive symptoms; therefore this medication should be prescribed cautiously for patients with high risk of depression. It seems that further studies would be necessary to determine behavioral effects of this medication in higher doses and in more susceptible patients.

## Background

Many cases of drug induced depression have been reported, specially amongst patients who are more susceptible for the disease. Drugs such as corticosteroids, Interferon Alpha, Angiotensin converting enzyme inhibitors and Calcium channel blockers are all apt to induce some depressive effects [1]. As depression and anxiety are important factors, concerning the quality of life and individuals' health [2], it is important to determine if any psychological or behavioral changes would occur with the prescription of a particular medication.

Finasteride, a potent 5alpha-reductase inhibitor, has been approved by Food and Drug Administration (FDA) in December 1997 for treatment of androgenetic alopecia in men [3]. The largest application of finasteride consists in treating benign prostate hyperplasia which is non-malignant enlargement of the prostate gland that leads to bladder out flow obstruction. In women finasteride has been used in some control trials for treatment of hirsutism [4].

Androgenetic alopecia or male pattern baldness is a common disorder affecting both men and women. However, the incidence is generally considered to be greater in male than female, and the pattern of hair loss differs between these two genders [5,6]. Finasteride, having a prominent therapeutic effect in men is used nowadays in a large scale for the treatment of the disease. Dose-ranging studies suggest the optimal dose of 1 mg per day. Whereas the patient may respond the treatment in a period of 3 to 4 months, but the treatment should be continued for at least 12 months before evaluating its complete efficacy [7]. In case of successful result, the medication is suggested to be used indefinitely for a long term. The disease begins to progress gradually when the treatment is stopped [3].

Pharmacologically, Finasteride inhibits 5alpha-reductase, the converting enzyme of testosterone to dihydrotestosterone (DHT) in many organs, such as prostate and hair follicles. Both androgenetic alopecia and benign prostate hyperplasia are androgen dependent disorders and associated with in situ high levels of intracellular DHT and increased 5alpha-reductase activity. As androgenic stimulation contributes to pathogenesis of both disorders, finasteride evokes a good therapeutic response by DHT suppression [8].

Steroid hormones, including androgens undergo an extensive metabolism in the brain. Several enzymes such as 5alpha-reductase, 3alpha-hydroxysteroid dehydrogenase (3alpha-HSD) and 17beta-hydroxysteroid dehydrogenase (17beta-HSD) intervene in brain androgen and steroid metabolism [9,10]. The substances synthesized by these enzymes affect not only sexual differentiation and behavior, but also non reproductive functions of the brain [11,12].

Animal studies suggested that finasteride could alter 5alpha-reductase activity in some regions of the brain, and lead to behavioral and mood changes. It has been shown that finasteride is able to inhibit 5alpha-reductase in medial basal hypothalamus in pregnant rats, and induce behavioral changes [13]. Significant inhibition of hypothalamic and pituitary 5alpha-reductase is also noticed in adult male rats [14]. In addition to animal studies, although there are some case reports suggesting finasteride induction of depressive symptoms and anxiety in humans, [15] but no prospective study has been carried out, in order to investigate finasteride behavioral effects. In the present study, we have investigated any depressive symptoms or anxiety induced by finasteride administration, in the patients with diagnosis of androgenetic alopecia.

## Methods

The study was approved by ethics committee of Tabriz University of Medical Sciences, Tabriz, Iran. The subjects were 20 to 38 years old men referred to Sina's dermatology clinic, a university affiliated educational center between January 2004 and December 2004. There was no age limitation for the subjects. Diagnosis of androgenetic alopecia was established by a dermatologist. After screening, 174 men who were prescribed finasteride 1 mg/day participated in a before-after study.

Patients with history of definitely diagnosed mood disorder and any chronic disease with exception of androgenetic alopecia (specially diseases which are associated with behavioral changes such as diabetes, hypothyroidism and asthma) were excluded from the study.

Patients using other medications at the beginning of treatment, and those who had used some in the period of 10 days before the study, were excluded as well. In addition, patients with history of medication usage for more than 21 days in the last three months before the study were not included in the investigation.

All subjects were explained the study process. In addition, all participants gave informed consent for the study. Contact information collected before the interview and during consent assignment. A standardized interview was used to obtain information on age, existence of any defined mood disorder or other diseases and history of medication use (systemic or topical).

Information on depressed mood was acquired using translated and validated Persian versions of Beck Depression Inventory (BDI) and Hospital Anxiety and Depression Scale (HADS) [16]. HADS translated and validated version is used for assessment of anxiety as well.

BDI has 21-item, self-administered inventory, and the score of each item ranges between 0 and 3. The score of ≤ 9 is considered to be within normal range, a score of 10–15 shows minimal depressive state, a score of 16–31 and 32–47 indicates mild and moderate depression respectively, and a score of > 47 exhibits severe depression.

HADS has 14-item, self-administered questionnaire. Seven items is related to anxiety, and the other seven, relates to depression. It has been shown that the HADS is a valid test not only in hospital practice, but in primary care and general population as well. Each question has 4 possible responses which are scored from 0 to 3. The two subscales, anxiety and depression have been found to independent measures [17]. Both anxiety and depression score is divided into four ranges: normal (0–7), mild (8–10), moderate (11–15), and severe (16–21).

First copy of BDI and HADS was completed by each participant at the beginning of the study, before finasteride treatment. The patients were informed about the fact that the effects of their medication would appear about 3 to 4 months or even more after the treatment onset, in order to avoid the depressive effects of apparent treatment failure in the first months. All patients were treated with finasteride 1 mg/day (Propecia 1 mg tablets, Merck Sharp & Dohme Ltd., UK), and asked to record if any other medication used in the period of the study in a diary.

The subjects were asked to complete both BDI and HADS questionnaires, two months after the treatment. At the same time, any extra medications, which had been recorded in the diary, was checked, and the patients were asked about any noticeable problems during the treatment. Patients showing with positive history of other medications were excluded also, except for those who had used Acetaminophen, Adult Cold, Aluminum Mg, and Aluminum MgS for less than 5 days.

The statistical analysis was performed by SPSS software version 11.5 (SPSS Inc. Chicago, IL). We used student's paired t test for comparing the means before and after the treatment. Mann-Whitney U test was used to compare the scores of patients who reported any side effects with others.

## Results

After interview, 144 eligible men were entered into the study. Nine patients excluded during the study because of having extra medication use, with seven others who refused to complete BDI or HADS.

One hundred and twenty eight men participated in the study completely. The mean age (± SD) was 25.8 ± 4.4 years. The mean BDI and HADS depression scores are listed in Table 1. According to the BDI scores at the beginning of the treatment, 46.9% of the subjects were within normal range, whereas 31.3%, 18.0% and 3.9% were within minimal depressive, mild depression and moderate depression range, respectively. There was no one within severe depression range.

**Table 1:** Comparison of BDI and HADS scores

	**Before treatment**	**After treatment**	**Sig.**
**BDI**	12.11 (± 7.50)	12.80 (± 7.64)	< 0.001
**HADS-D**	4.04 (± 2.52)	4.61 (± 3.19)	0.005
**HADS-A**	6.24 (± 3.17)	6.60 (± 3.06)	0.061

The mean ± SD of BDI score was 12.11 ± 7.50 and 12.80 ± 7.64 before and after the treatment, respectively. Analysis performed by paired t test, rejected the null hypothesis of no difference between the means after and before the treatment, and the BDI scores were significantly increased after treatment with finasteride 1 mg/day (p < 0.001). The 95% confidence interval, for the difference in the means of after and before treatment BDI scores, was ranging from 0.34 to 1.04.

We analyzed HADS depression scores as well. The mean ± SD of HADS-D score was 4.04 ± 2.51 and 4.61 ± 3.19, before and after the treatment. Analysis using paired t test, revealed that the HADS-D scores were also significantly increased 2 months after the treatment.

The mean ± SD of the HADS anxiety score was 6.24 ± 3.17 before the treatment which was increased to 6.60 ± 3.06 (Table 1), but the difference was not significant when means were compared using paired t test (p = 0.061). Based on the HADS anxiety scores at the beginning of the treatment, 60.9% of patients were within normal range, whereas 28.9% and 10.2% were within mild and moderate range, respectively. There was no one with severe anxiety, based on the HADS scores (Table 2).

**Table 2:** Individuals in different depression categories measured by BDI and HADS-A

**Depression categories**	**N**	**Percent**	**Mean (± SD)**
**Normal**	60	46.9	6.47 (± 2.14)
**Minimal depressive**	40	31.3	12.20 (± 1.70)
**symptoms**	23	18.0	22.26 (± 4.25)
**Mild depression**	5	3.9	32.60 (± 0.55)
**Moderate depression**	0	0	
**Severe depression**	0	0	

**Anxiety categories**	**N**	**Percent**	**Mean (± SD)**

**Normal**	78	60.9	3.99 (± 2.14)
**Mild anxiety**	37	28.9	8.95 (± 0.78)
**Moderate anxiety**	13	10.2	12.08 (± 0.95)
**Sever anxiety**	0	0	

Two month after the treatment, patients didn't report any side-effects, except for transient libido loss. Loss of libido was reported by 9.4% (12 patients) of the subjects. This was found to be transient and partial in all cases.

We compared BDI and HADS scores of the patients who complained of libido loss, with those who didn't report any side effects. Both before and after treatment scores compared by Mann-Whitney U test between two groups of libido loss positive and negative patients. No significant difference was found between the two groups (Table 3).

**Table 3:** Scores compared between two groups of libido loss positive and negative patients

	**Sig.**
BDI Before treatment	0.73
HADS-D Before treatment	0.61
HADS-A Before treatment	0.42
BDI After treatment	0.94
HADS-D After treatment	0.45
HADS-A After treatment	0.30

## Discussion

Testosterone, the main androgenic hormone, is converted by 5alpha-reductase to DHT in many tissues, which binds much more avidly with androgen receptor. Two isoforms of 5alpha-reductase with distinct tissue distribution have been cloned: 5alpha-reductase type1 and type2. 5alpha-reductase type1 is widely distributed in the body, whereas in androgen dependent structures such as prostate, and hair follicles of scalp DHT is mainly formed by 5 alpha-reductase type2. However, according to some studies this isoform is present in many other organs, such as pyramidal cells of cerebral cortex, hepatocytes and bile duct cells. [18,19].

After binding to the androgen receptor, DHT results in stimulation of transcription and protein synthesis. In spite of genomic response, the existence of cell membrane sex steroid receptors was also suggested recently. These receptors exist specially in the brain, and provoke rapid responses [20].

5alpha-reductase is a critical enzyme in the conversion of several steroids such as testosterone, progesterone, aldosterone and corticosterone in the brain. This enzyme converts testosterone to the most natural potent androgen DHT, and also it acts an important role in conversion of progesterone to dihhydroprogesterone (DHP). DHP is further converted to allopregnanolone (5alpha, 3alpha-tetrahydroprogesterone) by 3alpha-HSD [9,21]. Allopregnanolone is a modulator of gamma amino butyric acid type A receptor (GABA-A), and increases chloride conductance. This neurosteroid has been found to exert anti-convulsant, anesthetic and anxiolytic effects [22-24]. Moreover, change in the levels of allopregnanolone is found to be associated with depressive disorders. [25,26]

Our results are in agreement with the past published reports [15], and indicate that finasteride might induce depressive symptoms. The 95% confidence interval, for the difference in the means of the BDI scores, was ranging from 0.34 to 1.04. This shows that the overall change induced by finasteride is minimal, but statistically significant. Anxiety scores were also increased, but the difference was not significant.

A decline in serum DHT level occurs after finasteride administration [27]. This may contribute to finasteride induced depression. Some studies have been shown that serum DHT level, which is in equilibrium with the brain [28], is inversely associated with depression. A study by Barrett-Connor E. et al. showed that BDI scores were inversely associated with bioavailable testosterone and DHT level [29]. Furthermore, it was found that DHT had anti-depressant effects on behavior of male rats and its replacement in castrated rats was able to partially decrease the immobility behavior, which is indicative of depression [30].

Finasteride induced psychiatric dysfunction can also be attributed to its inhibitory effect on androgen and steroid 5alpha-reduction in the brain. Animal studies suggest that finasteride has inhibitory effects on 5alpha-reduction of testosterone and progesterone in the brain and inhibits the formation of allopregnanolone [31,32]. Allopregnanolone has an important role in depressive disorders [26]. In 1998 Romeo E. et al, revealed that episodes of unipolar major depressive disorder in men is associated with a decline in the plasma concentrations of allopregnanolone [25]. Furthermore, a study carried out by Uzunova V. et al. showed that CSF levels of allopregnanolone were significantly lower in depressed patients, and there was negative correlation between allopregnanolone levels in CSF and HAM-D scores. [33].

Since finasteride is a potent 5AR type2 inhibitor and the predominant isoform of the enzyme in human brain is 5AR type1 [34,35], some points should be noted concerning finasteride inhibitory effect on brain steroid metabolism. (i) Although finasteride is a potent 5AR type2 inhibitor, but it has also some inhibitory effects on 5AR type1 [36]. (ii) Finasteride administration in humans has been reported to be associated with some behavioral and mental disorders related to low levels of allopregnanolone in the brain [37]. This may also improve the concept of brain allopregnanolone suppression by finasteride in humans.

However, from the current study, it can not be concluded that the pathophysiology of finasteride-induced mood disorder is attributable to any of the mentioned material.

We couldn't find any significant change in the anxiety scores before and after the treatment (p = 0.061). According to the anxiolytic effects of allopregnanolone and testosterone [38-40], it seems that further investigations with a larger sample size are necessary to determine whether finasteride administration is associated with anxiety.

Finasteride associated side effects were reported by 9.4% of patients in this study. In all cases, the reported side effect was transient and partial libido loss. This side effect was reported by several studies and in different incidences [3,41]. Despite the fact that male sexual function and behavior is related to sex hormone metabolism [42], we couldn't find any relation between libido loss and BDI nor HADS score, before and after the treatment. This may be caused by the sample size effect as there were just 12 subjects in side effect positive group.

Some limitations concerning the study should be raised. The present study didn't have any control group. Excluding the subjects with definitely diagnosed other diseases or positive history of mood disorder, might result in missing some individuals with a high risk for behavioral changes. However, as a preliminary study, we had to exclude these patients to avoid any confounding effects on the results.

## Conclusion

In conclusion, this preliminary study suggests that finasteride might induce depressive symptoms. Although our data suggest a slight change, the side effect should be considered specially when the medication is prescribed for patients, who are more susceptible for it.

Finally, we recommend that further studies should be performed to elucidate behavioral effects of finasteride in higher doses and in high risk groups such as elderly patients.

## Competing interests

The author(s) declare that they have no competing interests.

## Authors' contributions

BR conceived of the study and participated in its design and the data analysis. RP performed selection of the participants and contributed to the data acquisition. PH participated in the data analysis. AM participated in the data acquisition and helped to draft the manuscript. All authors read and approved the final manuscript.

## Pre-publication history

The pre-publication history for this paper can be accessed here:


